# Analysis of the Relationships between Clinicopathologic Factors and Survival in Gallbladder Cancer following Surgical Resection with Curative Intent

**DOI:** 10.1371/journal.pone.0051513

**Published:** 2012-12-31

**Authors:** Xin-wei Yang, Jue Yang, Liang Li, Xiao-bo Man, Bao-hua Zhang, Feng Shen, Meng-chao Wu

**Affiliations:** Eastern Hepatobiliary Surgery Hospital, Second Military Medical University, Shanghai, People's Republic of China; Pontificia Universidad Catolica de Chile, Chile

## Abstract

**Background:**

This study elucidated the relationships between various clinicopathologic factors and the outcome of patients with gallbladder cancer (GBC) treated by surgical resection with curative intent.

**Methods:**

Between January 2003 and January 2011, 76 patients with GBC underwent surgical resection with curative intent at our department. We then conducted a retrospective analysis of clinicopathologic data. Fourteen clinicopathological variables were selected for univariate and multivariate analysis to evaluate their influence on the outcome.

**Results:**

The actuarial 1-, 3-, and 5-year survival rates in the 76 resected cases were 56.6%, 32.7%, and 23.8%, respectively. The univariate analysis revealed that curative resection (P<0.001), lymph node metastasis (P<0.001), AJCC stage (P = 0.030), tumor location (P = 0.008), histologic differentiation (P = 0.028), intraoperative blood loss (P = 0.011), and preoperative jaundice (P = 0.012) were significant risk factors for survival. Multivariate analysis revealed that noncurative resection and tumor location on gallbladder neck were significant risk factors for poor outcome. Among jaundiced patients, we discovered that gallbladder carcinoma with tumor thrombus in common bile duct (CBD) was very rare but with relatively special clinical manifestation and characteristic radiography manifestation. The prognosis of gallbladder carcinoma with tumor thrombus in CBD after surgical procedure was apparently better than gallbladder carcinoma with invasion of hilar tissues.

**Conclusions:**

Curative surgical resection remains the only effective approach to the treatment of GBC. This series confirm that jaundice is a poor prognostic factor. However, the presence of jaundice does not preclude resection, especially in highly selected patients (when R0 resection is achievable). Gallbladder carcinoma with tumor thrombus in CBD has special clinical characteristics, which need to be awared by radiologists and clinicians.

## Introduction

Gallbladder carcinoma has geographic and ethnic variation throughout the world and is a highly fatal malignant tumor. The poor prognosis of this disease is due to the anatomic position of the gallbladder and the nonspecific symptoms and signs [1], [2]. These characteristics of GBC result in advanced primary tumors and lymph node metastasis by the time of diagnosis. Because surgical resection is the only treatment that offers hope for cure, to elucidate the relationships between various clinicopathologic factors and the outcome of GBC patients treated by surgical resection with curative intent is very neccesary.

Several recent studies have shown that jaundice and extrahepatic bile duct involvement in gallbladder cancer are independent predictors of poor outcome [3]–[5]. Jaundice in gallbladder cancer usually results from cancer infiltration of the extrahepatic bile duct and indicates advanced disease [6], [7]. Many surgeons, especially those in Western countries, consider jaundice to be a contraindication to resection, despite the consensus that surgical resection offers the only chance of long-term survival [8], [9]. However, several experienced teams did not report such poor outcome and showed that some jaundiced patients obtained improved survival following resection of GBC [10]–[12]. A subgroup of jaundiced patients may therefore benefit from resection. This study was designed to re-assess the prognostic value of jaundice in GBC patients treated by surgical resection with curative intent.

## Patients and Methods

### Ethics

Written informed consent was obtained from all patients for surgical treatment and pathological examinations according to the institutional guidelines. All studys were approved by the Committee for the Ethical of Second Military Medical University.

### General information

A total of 76 GBC patients were treated with curative intent in our treatment goup at department of Biliary Surgery, Eastern Hepatobiliary Surgery Hospital, between January 2003 and January 2011. Their clinical characteristics, laboratory data, treatment including surgical procedure, operative findings, tumor pathological histology, operative outcome, and length of hospital stay were obtained from the database. Fourteen clinicopathological variables (age, sex, gallstones, preoperative jaundice, operative curability, location of tumors, AJCC [International Union Against Cancer, 7th edition] [13] pT factor, lymph node metastasis, UICC stage, histologic differentiation, hepatic invasion, pathologic extrahepatic bile duct invasion, intraoperative blood loss and djuvant therapy) were selected for univariate and multivariate analysis to evaluate their influence on the outcome.

### Criteria studied

R0 or R1 resections were considered to be resection with curative intent [12]. According to the UICC/AJCC TNM, 13 regional lymph nodes (gallbladder, pericholedochal, hepatic pedicle, proper hepatic artery and periportal nodes) were considered to be N1. Involvement of the periaortic, pericaval, superior mesenteric artery and/or celiac artery lymph nodes were classified as N2. Routine sampling of inter-aortocaval lymph nodes was not performed in this study. Postoperative hepatic insufficiency was defined by elevation of serum total bilirubin level >2.9 mg/dL and prothrombin time<50% persisting for more than 5 postoperative days [14].

### Surgical strategy

Our center's surgical policy for GBC is as follows: radical surgery for GBC. For radical surgery, partial hepatectomy with en bloc resection of the GB and dissection of regional lymph nodes (lymph nodes along the hepatoduodenal ligament and common hepatic artery and behind the pancreatic head) were routinely conducted. Partial hepatectomy includes extended right/left hepatectomy, right trisectionectomy or wedge resection with a 2-cm margin (including segments IVb/V). Combined resection of the bile duct, pancreas and/or duodenum was performed whenever direct invasion to these organs was suspected. If jaundice (serum bilirubin level more than 3 mg/mL) was identified preoperatively and postoperative hepatic insufficiency was highly suspected, percutaneous transhepatic biliary drainage (PTBD) or endoscopic retrograde biliary drainage (ERBD) was performed to reduce the cholestatic liver damage. For reduction of serum bilirubin levels or preoperative workup, PTBD and ERBD were performed for 3 and 4 patients, respectively.

### Demographic and clinical information

All 76 patients following surgical resection with curative intent were chosen as the subjects of the present study. The patients in whom hepatectomy and dissection of regional lymph nodes were expected to be curative on the basis of the preoperative imaging studies were candidates for surgery, and those in whom distant organ metastasis, cachexia, or extensive lymph nodal involvement was detected were considered not to have indications for hepatectomy. One patient underwent repeated hepatectomy for intrahepatic recurrence.

There were 26 men and 50 women, and their average age was 59 years (range 34 to 83). Sixty (78.9%) of the patients presented with some form of clinical manifestations, and the other 16 (21.1%) were asymptomatic. Fifty-five patients (72.4%) had associated liver disease: gallstones in 49, gallbladder polyp in 4 and chronic hepatitis (hepatitis B or C) in 2 (Table 1). Tumors diagnosed histologically after cholecystectomy are known as incidental GBC. In the study, among 76 patients with GBC referred to the hospital, in 19.7% of the patients (15 cases), the diagnosis of GBC has been missed at the time of routine cholecystectomy for gallstones in other hospital. In case of these incidental GBC, a radical second operation should also be offered to improve survival. The surgical procedures used to treat the patients are summarized in Table 2. In this study, “major hepatectomy” means right or left hepatectomy, extended right or left hepatectomy, or right or left trisegmentectomy; and “minor hepatectomy” means segmental resection or less. Of the total 77 hepatectomies in 76 patients, 4 (5.3%) were major, one of which was associated with combined caudate lobe resection, 1 with resection of the partial portal vein (PV) and one with hepatic artery resection. Combined resections of other organs were performed on 40 patients: bile duct resection (n = 32), pancreatoduodenectomy (n = 1), wedge resection of the duodenum (n = 1), segmental resection of the colon (n = 1) and partial gastrectomy (n = 5). Hepatoduodenal ligament lymph node dissection was performed routinely in all patients.

**Table 1:** Clinical characteristics of the 76 patients.

Items	Number (%)
Age (yr) average	59 (range 34–83)
Sex (M:F)	26:50
Body mass index(BMI)	22.6 (range 14.8–29.8)
Clinical presentation	
Asymptomatic	16 (21.1%)
Symptomatic*	60 (78.9%)
Associated gallbladder/liver disease	
Hepatitis B or C positive	2(2.6%)
gallstones	49(64.5%)
gallbladder polyp	4(5.3%)
Nil	21(27.6%)
Incidental GBC	15(19.7%)

* Symptoms: jaundice, abdominal discomfort, general fatigue, abdominal mass.

**Table 2:** Types of 77 hepatectomy performed in the 76 gallbladder cancer patients including a repeat hepatectomy.

Treatment	Number
Extent of liver resection	
Major hepatectomy(>3 segments)	4
Anatomical segments IV-V	22
Gallbladder bed	50
Left hepatectomy*	1
Other procedures	
Lymphadenectomy	76
Portal vein resection	1
Hepatic artery resection	1
Common bile duct resection	32
Adjacent organ resection	
Colectomy	1
Gastric resection	5
Pancreaticoduodenectomy	1
Duodenal resection	1
Vascular clamping	60(11.4min, range 4–20min)
Intra-operative bleeding(ml)	Mean 586(range 200–3200ml)
Operative time(min)	Mean 258.6(range 90–470min)
R0	58(76.3)

* Includes a patient with repeated hepatectomies for intrahepatic recurrence.

The surgeons assisted the pathologists to correctly identify complicated resection margins during preparation of sections of the fixed specimens. The surgical resection was considered curative, if all pathologic margins were free of tumor and there were no residual tumor. Well or moderately differentiated adenocarcinoma was diagnosed in 82.9% of the tumors and poorly differentiated adenocarcinoma in the other 17.1%. Stage grouping in the present study was performed according to the system of the pTNM classification of the International Union Against Cancer (UICC), 7th edition [13]. Most of the patients (82.9%) had UICC stage III or IV lesions at the time of diagnosis and treatment. Lymph node metastases (45 cases), hepatic invasion (40 cases), vascular invasion (2 cases) and involving the extrahepatic bile duct (31 cases) were recognized pathologically in 59.2%, 52.6%, 2.6%, and 40.8%, respectively, of the cases (Table 3). Twenty-three patients received adjuvant therapy: intraoperative and postoperative chemotherapy in 11 patients, postoperative radiotherapy in 9, and a combination of chemotherapy and radiotherapy in 3.

**Table 3:** Pathological characteristics of the 76 GBC.

Pathological classification	Number (%)
Histologic type	
Well differentiated	3 (3.9)
Moderately differentiated	60 (79.0)
Poorly differentiated	13 (17.1)
Tumor location	
gallbladder neck	31 (40.8)
gallbladder body	24 (31.6)
gallbladder fundus	21 (27.6)
Tumor extension	
Hepatic invasion	40 (52.6)
Lymphatic invasion	45 (59.2)
Extrahepatic bile duct invasion	31 (40.8)
Vascular invasion	2(2.6)
Stage (UICC)	
Early (stage I and II)	9 (11.8)
Advanced (stage III, IV)	67 (88.2)

### Statistical analysis

Non-parametric data was presented as median (range) and categorical data was presented as frequency and proportion (%).Variables were compared by the χ^2^ test, Fisher's exact test or Mann-Whitney's U test, where appropriate. Overall survival was measured from the day of operation to death, including death due to cancer or to other causes, and to the last day of follow-up. Follow-up was continued until July 28, 2012, or until death, if earlier. Survival rates were estimated by the Kaplan-Meier method, and the differences between survival curves were tested by the log-rank test. A P value <0.05 was considered significant. The multivariate analysis was performed according to Cox's hazard model.

## Results

### Morbidity, mortality, and overall survival rates

The mean duration of the postoperative hospital stay was 14.6 days (range 8 to 85). The postoperative morbidity and mortality rates were low even after major hepatectomy with combined resection of adjacent organ or extrahepatic bile duct. There was only one hospital death, who died on postoperative day 19 due to multiple organ failure (including hepatic failure) secondary to intra-abdominal infections after major hepatectomy and choledochojejunostomy. Morbidity occurred in 18 of the 76 ICC patients (23.7%), which was consisted of intra-abdominal infections in 2 patients, seroperitoneum needing to puncture in 9 patients, abdominal bleeding in 2 patients, bile leakage in 3 patients,liver abscess in 1 patient,and incision infection in 1 patient.

**Figure 1:**
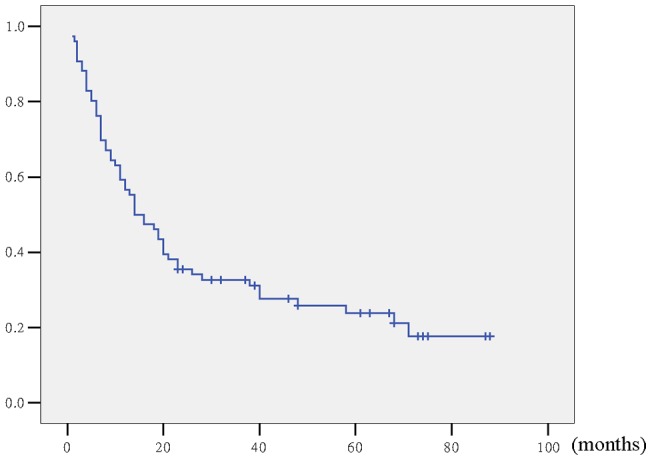
Actuarial survival curve of 76 gallbladder cancer patients following surgical resection with curative intent.

The median follow-up time was 23.9 months. There was no patient lost in follow-up. The median survival time of the entire 76 patients followed up was 14.0 months (range 1.0 to 88). The actuarial 1-, 3-, and 5-year survival rates of all 76 patients were 56.6%, 32.7%, and 23.8%, respectively (Fig. 1).

### Univariate analysis

Univariate analysis revealed that neither age, sex, gallstones, pT factor, hepatic invasion, pEBI during which they initially examined, nor adjuvant therapy were significant factors for survival. By contrast, curative resection (P<0.001), lymph node metastasis (P<0.001), AJCC stage (P = 0.030), tumor location (P = 0.008), histologic differentiation (P = 0.028), intraoperative blood loss (P = 0.011), and preoperative jaundice (P = 0.012) were found to be significant risk factors for survival (Table 4).

**Table 4:** Univariate analysis of 14 variables in relation to survival (76 cases).

			Survival rates (%)		
Variables	Cutoff levels	Number	3 year	5 year	P value
Age (yr)	59	41	23.4	17.6	0.061
	≤59	35	42.9	31.4	
Sex	Male	26	30.3	22.7	0.976
	Female	50	33.9	21.1	
Jaundice	Present	27	14.8	7.4	0.012
	Absent	49	42.7	34.0	
Associated gallstone	Present	48	32.8	21.5	0.639
	Absent	28	32.1	27.6	
Curability	Curative	58	41.1	29.2	<0.001
	Noncurative	18	5.6	5.6	
Tumor location	Gallbladder neck	31	19.4	7.7	0.008
	Gallbladder body/fundus	45	42.0	36.0	
pT (UICC)	pT1 and 2	18	44.4	37.0	0.119
	pT3 and 4	58	28.9	19.8	
Lymph node metastasis	Negative	31	57.3	53.3	<0.001
	Positive	45	15.6	5.2	
Stage(UICC)	I and II	13	53.8	35.9	0.030
	III and IV	63	23.1	17.5	
Histologic differentiation	Well/Moderate	63	34.6	28.1	0.028
	Poor	13	23.1	0	
Hepatic invasion	Present	40	27.3	18.2	0.126
	Absent	36	38.7	29.5	
pEBI	Present	31	19.4	11.6	0.056
	Absent	45	42.0	32.7	
Intraoperative blood loss	≤600ml	57	36.5	29.6	0.011
	>600ml	19	21.1	7.0	
Adjuvant therapy	Yes	23	39.1	33.5	0.151
	No	53	27.4	19.6	
	Overall	76	32.7	23.8	

pEBI indicates pathologic extrahepatic bile duct invasion.

### Multivariate analysis

A multivariate analysis was performed to determine which univariate prognostic relationships were independent predictive factors (Table 5). The results are shown in Table 5. Noncurative resection and tumor location on gallbladder neck were found to be significant risk factors for poor outcome.

**Table 5:** Results of multivariate analysis.

Variable	Regression coefficient	Standard error	*P* value	Relative risk	95% Confidence interval
Jaundice	0.25	0.34	0.463	1.284	0.659–2.500
Curability	1.189	0.356	0.001	3.285	1.636–6.597
Tumor location	−0.733	0.323	0.023	0.480	0.255–0.904
Stage(UICC)	0.423	0.553	0.444	1.527	0.516–4.519
Lymph node metastasis	0.68	0.440	0.122	1.974	0.833–4.674
Histologic differentiation	0.468	0.372	0.209	1.597	0.770–3.313
Intraoperative blood loss	0.496	0.314	0.114	1.642	0.887–3.038

### Clinicopathologic Features of Nine 5-Year Survivors

Of the 76 patients, 9 survived more than 5 years. There were 2 male and 7 female survivors with an average age of 57.1 years. None presented with jaundice. All the patients had been treated by removal of the GB, wedge resection of the GB bed (including segments IV and V) and portal lymphadenectomy. There was no combined resection of adjacent organs. Curative resection was obtained in all of them. Histologically, none of the patients had lymph node metastasis. Seven of the 9 patients were still alive without tumor recurrence. Of the other 2 patients, one died of tumor recurrence at 5 years 11 months, and the other died of cardiovascular disease disease at 5 years 8 months.

### Clinicopathologic Features of the 27 Jaundiced Patients

**Figure 2:**
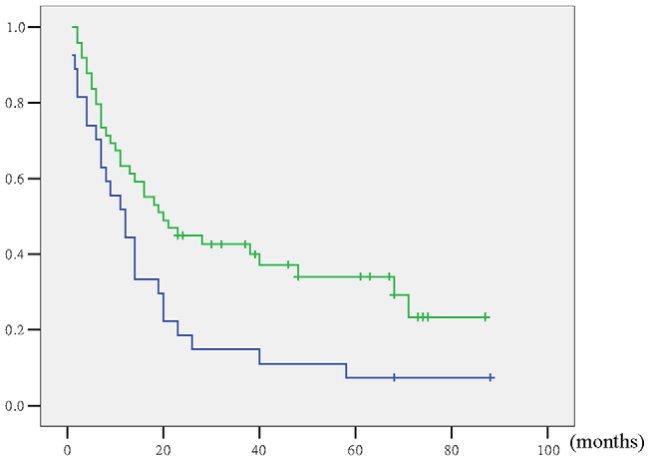
Actuarial survival curve according to preoperative jaundice. (With versus without jaundice: P = 0.012.).

The clinicopathologic features of the 76 patients, grouped according to presence or absence of preoperative jaundice, were summarized in Table 6. All patients with preoperative jaundice underwent extrahepatic bile duct resection and reconstruction with curative intent. Combined resection of adjacent organs was necessary in more patients with preoperative jaundice. A longer postoperative hospital stay and more lymph node metastasis were associated with preoperative jaundice. As a result, R0 resection was more difficult to obtain in patients with preoperative jaundice than those without preoperative jaundice. Five-year survival rate and median survival time for the 49 patients without preoperative jaundice was 34% and 20.0 months, and for the 27 patients with preoperative jaundice were 7.4% and 12.0 months. Survival for patients with preoperative jaundice was significantly worse than for patients without preoperative jaundice (P = 0.012) (Fig. 2), though was not significant risk factor in multivariate analysis (P = 0.463).

**Table 6:** Demographic data of jaundiced (n = 27) and non-jaundiced patients (n = 49) with gallbladder cancer.

Variables	Jaundiced, n	Non-jaundiced,n	p-Value
Male gender	12	14	0.166
Mean age (range)	58.3(35–78)	59.4(34–83)	0.858
Postoperative hospital stay	19.6(8–85)	11.9(8–33)	0.001
Extent of liver resection			0.330
Major hepatectomy(>3 segments)	1	3	
Anatomical segments IV-V	7	17	
Gallbladder bed	19	29	
Combined resection of adjacent organs	7 (25.9%)	2 (4.1%)	0.005
Microscopic invasion of theliver parenchyma	18 (66.7%)	22 (44.9%)	0.071
Lymph node metastasis	22 (81.5%)	23 (46.9%)	0.004
pT			0.091
pT4	9 (33.3%)	9 (18.4%)	
R0	17 (63.0%)	41 (83.7%)	0.043
Mortality (number of patients)	9(33.3%)	9(18.4%)	0.145

Note that adjacent organs include the pancreas, duodenum, stomach, and/or colon other than the liver and extrahepatic bile duct.

### An unusual way of growing invasion in GBC

**Figure 3:**
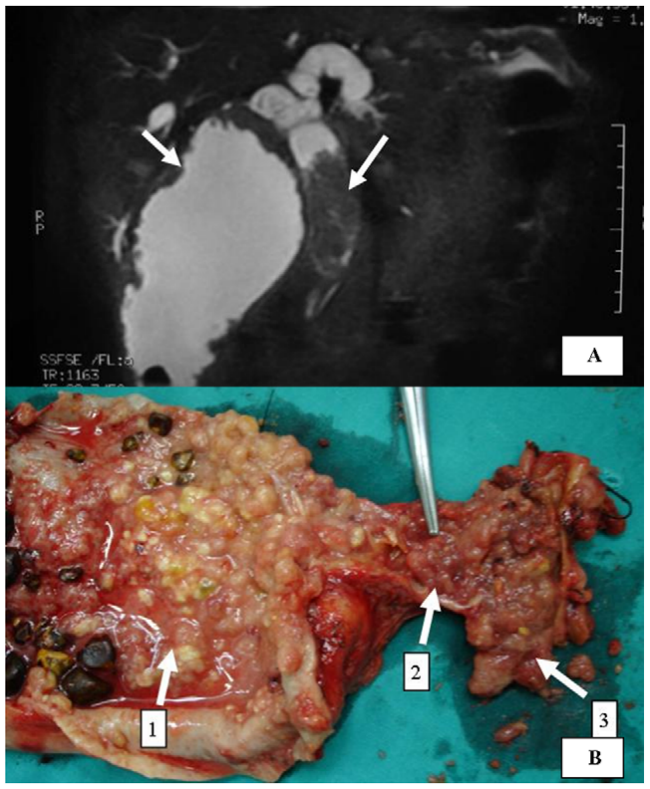
A jaundiced gallbladder carcinoma with tumor thrombus in common bile duct. (A: MRCP photography shows filling defect in CBD and gallbladder; B: In surgery specimen arrows 1–3 point at tumor tissues in gallbladder, cystic duct and CBD respectively.) We state that the subject of the photograph has given written informed consent by the patient to publication of the photograph.

In this period, three patients with gallbladder carcinoma who were identified of tumor thrombus in common bile duct in surgical procedure were analyzed. Abdominal ultrasound and magnetic resonance cholangiopancreatography (MRCP) were used for preoperative diagnosis (Fig. 3). All 3 patients were given radical operations, which were composed of cholecystectomy, resection of the extrahepatic biliary duct, cuniform hepatectomy of gallbladder bed, skeletonization of the hepatoduodenal ligament, hilar choledochojejunostomy and clearance of tumor thrombus from bile duct. All three patients were recovered well after surgery, which were respectively alive for 30 months, 17 months and 23 months without tumor recurrence, and 58 months, 41 months and 40 months for survival time after operation. Gallbladder carcinoma with tumor thrombus in common bile duct was very rare but with relatively special clinical manifestation and characteristic radiography manifestation. MRCP was one of the most potent diagnostic method. The prognosis of gallbladder carcinoma with tumor thrombus in common bile duct after surgical procedure was apparently better than gallbladder carcinoma with invasion of hilar tissues. Radical operation was feasible and safe for obtaining longer survival.

## Discussion

The majority of the patients with GBC remain asymptomatic or have vague complaints in the early stage of the disease, and by the time they become symptomatic, the tumor is in the advanced stage [1]–[4]. In the present study, we found that whereas 21.1% of the patients were asymptomatic, 78.9% had some form of symptoms, including abdominal discomfort, abdominal pain, abdominal mass, or jaundice at the time of diagnosis. Because of this unfortunate state of affairs, the prognosis of GBC is still poor despite improvements in diagnosis and therapy. The median survival time in our 76 cases was 14 months, and the overall actuarial 1-, 3-, and 5-year survival rates were 56.6%, 32.7%, and 23.8%, respectively. These values are comparable to those in the literature on the treatment of GBC [1]–[3], [8], [12].

Many investigators have used multivariate analysis to determine useful prognostic factors for gallbladder carcinoma after surgical resection. According to these reports, potentially significant factors include nodal involvement [12], [15]–[17], hepatic invasion [16], [18], choledochal invasion [3], [16], [18], pathological grading of differentiation [17], [19], vascular invasion [20], and pathologically curative resection [3], [12], [17], [20], [21]. In the current study, multivariate analysis revealed that noncurative resection and tumor location on gallbladder neck were independent prognostic factors.

Gallbladder neck is in an anatomically “busy” area due to presence of adjoining bile duct, portal vein, liver, duodenum and colon, which become involved early, making surgical resection and radiotherapy difficult [22]. Because of the particularly anatomical location, gallbladder neck tumors greatly increased the difficulty of the surgery and reduced the opportunities for radical resection. At the same time, a relatively small tumor in the gallbladder neck infiltrates the hepatic hilum and causes obstructive jaundice [23]. In our study, we also found that survival was significantly worse in jaundiced patients than non-jaundice, simultaneously with lower R0 resection rate in jaundiced patients (P = 0.043).

With recent developments in imaging modalities, precise preoperative diagnosis promotes the performance of extended right hemihepatectomy in jaundiced patients with gallbladder carcinoma as a means of achieving curative resection. However, the major obstacle to this procedure has been a high incidence (13–27 percent) of postoperative mortality from hepatic failure [24]. The recent application of preoperative biliary drainage and PVE has made it possible to perform this procedure safely with a low mortality rate [25], [26]. In the present study, 7 patients in 27 jaundiced patients underwent preoperative biliary drainage. Hepatic failure occurred in only one patient. Firstly, one reason for the lack of mortality may be a lower frequency of additional major surgical procedures, such as pancreaticoduodenectomy (only 1 of 76 patients, 1.3 percent). Considering the high risk of death and poor longterm survival, there is no definitive merit of combined major hepatectomy and pancreaticoduodenectomy for jaundiced patients with gallbladder carcinoma [27]. It has been reported that the extent of hepatectomy is not related to R0 rate and long-term survival [28]. Secondly, a parenchymal preservation strategy must be investigated in this setting in the light of the results published by Agarwal et al.: in this series of 14 resected jaundiced patients, 13 patients underwent segment IVB-V liver resection with a very low (one patient) mortality, 100% R0 resection rate, and meaningful prolongation of survival [10]. A similar strategy of minor resection has been recently recommended by Regimbeau for jaundiced patients with gallbladder cancer [12]. In this study, we have retained the most normal liver parenchyma, to reducing the incidence of postoperative complications, on the premise of R0 resection. We also have got a comparable five-year survival rate. Therefore, we recommend minor resection when negative margin was ensured in operation.

### Jaundice should not be considered as an absolute contraindication

Most studies have reported that jaundice is an indicator of advanced disease with a dismal prognosis in GBC [1], [3], [7], [10]–[12]. In the present study, we confirmed that jaundiced patients had longer postoperative hospital stay and poorer 5-year survival than non-jaundiced patients (7.4% and 34%, respectively), even though preoperative jaundice was not a significant risk factor for poor outcome in multivariate analysis.

Therefore, we have not considered jaundice alone to be a contraindication to resection. Some jaundiced patients appeared to benefit from surgery in terms of survival following resection. Moreover, the extent of bile duct invasion, rather than jaundice, is a determinant of resectability due to anatomical reasons. In this study, preoperative jaundicewere found to be a significant risk factor for survival in univariate analysis (P = 0.012). However, pEBI was not a significant risk factor(P = 0.056). This means pathologic extrahepatic bile duct invasion does not always accompany jaundice. Strictly speaking, these two forms of GBC infiltration should be distinguished in further study. In conclusion, advanced gallbladder cancer with extrahepatic bile duct invasion and/or jaundice is a candidate for resection when R0 resection is achievable. However, radical resection of such advanced gallbladder cancer is still challenging.

### A special infiltration manner in jaundiced patients with GBC

To date, only Midorikawa et al. [29] described one case of tumor embolus in the CBD from gallbladder carcinoma; however, the tumor embolus he described was separated from the tumor. According to our own cases and literature review, gallbladder carcinoma with tumor thrombus in the CBD has the special clinicopathologic characteristics and better prognosis. GBC with cancer embolus extending into the CBD has different imaging manifestations on MRCP from GBC infiltrating the hilar bile duct. The latter usually displays abrupt truncation of the extrahepatic bile duct on MRCP. In contrast, GBC with cancer embolus in the CBD manifested that the dilation of extrahepatic and intrahepatic bile ducts was lacked of asymmetry. While GBC with tumor thrombus in the CBD usually developed intraductally, infiltration of liver was uncommon. In three cases, postoperative pathology revealed that all three patients were only invaded to muscular layer.

It has been reported that advanced gallbladder carcinoma with obstructive jaundice has a poor prognosis, and the advantages of radical surgery for these patients are still controversial [3], [7], [10]–[12], [22]. Observing our own patients and the patient reported by Midorikawa [29], since obstructive jaundice caused by tumor thrombus is not always associated with advanced staging, radical surgery should be performed. The prognosis of gallbladder carcinoma with tumor thrombus in the CBD after radical surgery maybe apparently better than gallbladder carcinoma with invasion of hilar tissues.

In conclusion, curative surgical resection remains the only effective approach to the treatment of GBC. Extensive resection is indicated if lymph node metastasis can not be identified preoperatively or intraoperatively. This series confirms that jaundice is a poor prognostic factor. However, the presence of jaundice does not preclude resection, especially in highly selected patients. Among jaundiced patients, gallbladder carcinoma with tumor thrombus in the CBD had the different clinical, radiological, and prognosis characteristics, which need to be awared by radiologists and clinicians as a special type of gallbladder carcinoma.

However, the limitations of this study are its retrospective design and the small number of patients studied. Further studies on larger numbers of patients, including prospective studies, are required to confirm the results of this study.
